# The systemic evolutionary theory of the origin of cancer (SETOC): an update

**DOI:** 10.1186/s10020-025-01069-w

**Published:** 2025-01-14

**Authors:** Antonio Mazzocca, Giovanni Ferraro, Giovanni Misciagna

**Affiliations:** 1 Interdisciplinary Department of Medicine, University of Bari School of Medicine, Piazza G. Cesare, 11, 70124 Bari, Italy; 2 Association for Systems Science, Via S. Stefano, 42, I-75100 Matera, Italy

**Keywords:** Cancer theories, Endosymbiosis, Archaea, Evolution, Systems biology, Mitochondria, Warburg effect

## Abstract

The Systemic Evolutionary Theory of the Origin of Cancer (SETOC) is a recently proposed theory founded on two primary principles: the cooperative and endosymbiotic process of cell evolution as described by Lynn Margulis, and the integration of complex systems operating in eukaryotic cells, which is a core concept in systems biology. The SETOC proposes that malignant transformation occurs when cells undergo a continuous adaptation process in response to long-term injuries, leading to tissue remodeling, chronic inflammation, fibrosis, and ultimately cancer. This process involves a maladaptive response, wherein the 'endosymbiotic contract’ between the nuclear-cytoplasmic system (derived from the primordial archaeal cell) and the mitochondrial system (derived from the primordial α-proteobacterium) gradually breaks down. This ultimately leads to uncoordinated behaviors and functions in transformed cells. The decoupling of the two cellular subsystems causes transformed cells to acquire phenotypic characteristics analogous to those of unicellular organisms, as well as certain biological features of embryonic development that are normally suppressed. These adaptive changes enable cancer cells to survive in the harsh tumor microenvironment characterized by low oxygen concentrations, inadequate nutrients, increased catabolic waste, and increased acidity. De-endosymbiosis reprograms the sequential metabolic functions of glycolysis, the TCA cycle, and oxidative phosphorylation (OxPhos). This leads to increased lactate fermentation (Warburg effect), respiratory chain dysfunction, and TCA cycle reversal. Here, we present an updated version of the SETOC that incorporates the fundamental principles outlined by this theory and integrates the epistemological approach used to develop it.

## Introduction

Cancer is described as a disease caused by genetic mutations in somatic cells due to the mutagenic or genotoxic action of carcinogens. This represents the basis of the somatic mutation theory (SMT), which is the dominant paradigm for carcinogenesis, a paradigm that is seen as a fact rather than a theory. The SMT has recently faced opposition from alternative ideas of the disease. One such theory is the Tissue Organization Field Theory of Cancer (TOFT), proposed by Sonnenschein and Soto (Sonnenschein and Soto 2005), which views cancer as a disease of tissue communication. The metabolic theory is a different theory proposing that cancer originates from mitochondrial dysfunction, which was first introduced by Otto Warburg and later expanded upon by Thomas Seyfried (Seyfried et al. 2015). These divergent perspectives, like many scientific theories, have sparked significant controversy, but they have not received the attention they deserve, probably because they challenge the SMT. Questioning the current paradigm could potentially undermine the effectiveness and adequacy of many existing pharmacological treatments for cancer that are based on SMT. Our previously proposed Systemic Evolutionary Theory of Cancer (SETOC) provides a conceptual framework that integrates earlier theories and may facilitate the development of innovative cancer treatment strategies (Mazzocca et al. 2016, 2018; Mazzocca 2019; Mazzocca et al. 2021). Nearly a decade later, we present an updated version of this theory.

### Evidence against the somatic mutation theory (SMT)

Over the years, a significant body of scientific evidence has revealed the weaknesses of SMT, including the following:

1. Mutations that activate oncogenes and inactivate tumor suppressor genes, hallmarks of certain types of tumors (such as those found in the esophagus and skin), have been found in corresponding normal tissues (Marticonrena et al. 2015; Marticonrena et al. 2018; Chanock 2018).
2. The Cancer Genome Atlas (TCGA) project identified hundreds of random mutations in various forms of cancer. Every cancer has numerous random mutations; however, a specific signature for any tumor has not yet been discovered.
3. In nuclear transplantation experiments, when tumor cell nuclei are transplanted into normal mouse blastocysts, tumor-free embryos and mice develop. Conversely, tumors form when healthy cell nuclei are transferred into the cytoplasm of tumor cells (Darlington et al. 1937; Brinster et al. 1974; Mintz et al. 1975).
4. Several sequencing studies have reported that some tumors have zero mutations in their DNA, particularly in the coding exons (Greenman et al. 2007; Lawrence et al. 2013).
5. If targeted therapies, based on the idea that cancer is a genetic disease, were effective, they would have replaced chemotherapy and radiation by now. Disappointing clinical results suggest that the SMT does not fulfill its expected role (Prasad et al. 2016; Barone et al. 2020).
6. The dramatic increase in cancer incidence in both the Western and Eastern world, which have adopted a Western lifestyle, suggests that cancer is not a genetic disease (Bray et al. 2024).
7. Not all mutations found in tumors are procarcinogenic. It is difficult to imagine that every identified mutation or its overall impact causes cancer. Some mutations are protective. This is the case for mutations in the IDH1 gene, which are associated with a better prognosis in patients with glioma (Sanson et al. 2009).

### Hypothesis: epistemology of the SETOC

#### An ontological perspective on cancer

From an ontological perspective, cancer is a complex subject. On one hand, the disease is characterized by various forms; on the other hand, specific biological traits, known as the Hallmarks of Cancer, are shared among different tumors. These characteristics were detailed in two influential articles by Hanahan and Weinberg (Hanahan and Weinberg 2000, 2011), among the most cited works in the field of tumor biology. Different types of tumors, such as chronic myeloid leukemia and hepatocellular carcinoma, exhibit biological commonalities as outlined by the Hallmarks of Cancer. Although tumors are highly heterogeneous, displaying variations across different types, among individuals, and even within a single tumor, cancer cells nonetheless share common characteristics and principles rooted in biological mechanisms. This understanding may facilitate the development of a unified cancer treatment strategy. While the Hallmarks of Cancer possess significant educational value, they are fundamentally descriptive in nature and do not clarify the dynamics of the disease, particularly its pathophysiology. Furthermore, they do not provide insight into the primary etiological factors that contribute to cancer.

#### A theoretical framework provides a foundation for understanding the SETOC

A scientific theory is a model or a set of models that explains available observational data and provides testable predictions. In physics, theories often manifest as laws and are identified through inductive reasoning. In contrast, theories in biology exhibit a significant degree of complexity, frequently taking the form of concepts and mechanisms. These biological theories are typically developed through the process of abductive reasoning. As noted by Ernst Mayr, “concepts” are fundamental to the discipline of biology (Mayr 2004). Unlike physics, which adheres to strict laws, biology relies on concepts such as evolution, speciation, biodiversity, and reproduction. However, when examining the physiological functions of living organisms from a strictly mechanistic perspective, these functions can ultimately be linked to the fundamental principles of physics and chemistry. Regrettably, many cancer theories have been formulated from a physics perspective, often treating these theories as if they were definitive laws. For instance, the notion that genes are the primary drivers of cancer development aligns more closely with a physical interpretation than with a biological understanding. The premise of the SMT is that cancer arises solely from mutations in oncogenes or tumor suppressor genes; if no mutations occur, cancer does not develop. However, we now understand that oncogenes and tumor suppressor genes are neither necessary nor sufficient causes of cancer (Sonnenschein and Soto 2018). Probabilistic causal approaches provide limited insight, as altered genes identified in cancer are also found in normal tissues (Marticonrena et al. 2015; Marticonrena et al. 2018; Chanock 2018). Furthermore, in the context of cancer research, the advancement of knowledge through inductive reasoning has been both complex and convoluted. This situation primarily arises because the typical cause-and-effect relationship has been inverted: first, the effect (i.e., the tumor) is identified, and then the cause (i.e., the mutated gene) is determined through case-series studies, which are considered to have the lowest level of validity among epidemiological study methodologies. If we had accurately analyzed causality, we would have discovered that mutated genes are not exclusive to tumors; rather, they are also present in many normal tissues that do not develop cancer over time (Marticonrena et al. 2015; Marticonrena et al. 2018). It would have been beneficial to compare tumor tissue with adjacent nontumor tissue to identify mutated genes, as is commonly practiced in epidemiological case–control studies. Paradoxically, the comparison was conducted only after the SMT had become the dominant framework for explaining cancer (Chanock 2018). In biology, mechanisms are essential for elucidating the functions of biological structures. In cancer biology, identifying a comprehensive mechanism that accounts for the hallmarks of cancer is critical. Such a mechanism may include genetic alterations but should not rely solely on cancer genetics. More precisely, this mechanism involves a comprehensive process of cellular adaptation to external factors, which includes not only genetic changes but also various other cellular mechanisms, rather than being driven exclusively by genetic factors. The SETOC provides a general mechanism (epistemologically developed through abductive reasoning) to interpret carcinogenesis and the hallmarks of cancer. This mechanism, termed “de-endosymbiosis” (see below), is initiated by chronic insults of various types, leading to the functional decoupling of cellular subsystems and energy dysfunction in eukaryotic cells.

#### The SETOC proposes a non-Darwinian mechanism of evolution for the development of cancer

According to contemporary perspectives, cancer progresses through a Darwinian process of natural selection, where the most resilient tumor cell populations thrive and are preferentially selected. Specifically, in cancer, there is a selection process whereby neoplastic cells outcompete normal cells and more aggressive neoplastic cells supersede less aggressive tumor cells. The SETOC proposes an alternative evolutionary framework for understanding cancer that diverges from traditional Darwinian evolution and instead draws on Margulian cellular evolution (Sagan 1967; Margulis 1970, 1981, 1998). The SETOC proposes a cellular adaptive mechanism, called ‘de-endosymbiosis,’ which is based on the endosymbiosis that led to eukaryogenesis and explains how neoplastic cells can form long before Darwinian selection influences their progression. According to the SETOC, neoplasms originate at the cellular level within a tissue as a response to forced adaptation or maladaptation. When cells experience maladaptation, the two cellular endosymbiotic systems—the nucleocytoplasm and mitochondrion—decouple, thereby affecting the fundamental biological mechanisms that have driven the evolution of eukaryotic cells.

#### The SETOC is a fundamental theoretical framework of systems biology for understanding cancer

The functions of biological systems, including cells, are currently being studied via systems theory. This approach is also utilized to investigate the behavior of cancer cells. While the systemic approach to normal cells places a strong emphasis on networks, it often overlooks the organizational level of the cell, particularly the cooperative functioning of the 'subsystems' within eukaryotic cells. This oversight has significant implications for our understanding of neoplastic cell function. The SETOC investigates how the interactions between cellular subsystems that originally led to the emergence of eukaryotic cells (eukaryogenesis) also contribute to cancer through their uncoordinated dysregulation. This process ultimately results in the expression of functions and processes akin to those observed in single-celled organisms (phylogeny) and embryos (ontogeny). The SETOC provides a dynamic mechanism that enables cells to adapt to prolonged external stressors, creating positive feedback that drives them through transitional phases triggered by de-endosymbiosis. During de-endosymbiosis, the two subsystems decouple and become uncoordinated, allowing the archaeal core to take control and impart its inherent ability to proliferate to the entire cell. As François Jacob famously stated, “The dream of every cell is to become two cells.”

### Evaluation of the hypothesis: fundamentals of the SETOC

The theoretical foundation of SETOC does not focus on altered genes but on the comprehension of eukaryotic cell functions through the frameworks of systems biology and evolution. Eukaryotic cells are believed to have originated from the endosymbiosis of two prokaryotic species: an archaeon, which served as the engulfing cell that formed the nucleus and cytoplasm, and an α-proteobacterium, which evolved into the mitochondrion with a significant portion of its DNA transferred to the nucleus (Lane and Martin 2010). Compelling evidence supports the notion that the host cell for eukaryotes was indeed an archaeon (Williams et al. 2013; Zaremba-Niedzwiedzka et al. 2017; Spang et al. 2018). In mammalian cells, energy production primarily occurs through the integration of glycolysis in the cytoplasm and the TCA cycle and oxidative phosphorylation (OxPhos) within the mitochondria. From a biology systems standpoint, mitochondria function in series with the cytoplasm and in parallel with each other. Glycolysis precedes the TCA cycle, forming a linear sequence of biochemical reactions. Simultaneously, the TCA and OxPhos independently within the mitochondria. After each glycolytic process, a limited amount of ATP is produced, contributing to the cell´s energy. To generate more ATP in a single complete process, pyruvate (the end product of glycolysis) must enter the TCA cycle. This cycle operates in a forward mode and is coupled with OxPhos. The energy requirements of differentiated eukaryotic cells depend on the entire process. Glucose is converted to pyruvate, which then enters the mitochondria and is transferred to the TCA cycle. This cycle operates in a forward mode, converting pyruvate into water (H_2_O) and carbon dioxide (CO_2_) in the presence of oxygen. H_2_O and CO_2_ are subsequently eliminated from the body through respiration and urine. In eukaryotes, the disposal of metabolic waste products is essential, as these byproducts can be recycled for cellular sustenance and division. The integration of glycolysis, the TCA cycle, and OxPhos, along with the optimization of energetic investments in eukaryotic cells, was likely necessary to better process waste products during metabolism. This ultimately leads to the emergence of multicellular organisms during evolution by promoting cell aggregation (Pfeiffer and Bonhoeffer 2003; Lynch 2024). If waste products are not properly eliminated, the metabolism of the cells may be impaired, leading to a dysplastic or neoplastic state characterized by uncontrolled cell duplication, dedifferentiation, and eventually migration from neighboring cells. The concept of cellular transitional states is well exemplified by dysplasia. Dysplastic cells can redirect their lineage away from their origin, much like multipotent embryonic cells. This ability allows cells to execute programs that help them cope with harsh microenvironmental conditions. In an altered microenvironment, cells enter a transitional state where specific cellular programs and gene expression are crucial, and genetic mutations are a secondary consequence of the chaotic proliferative state (Prehn 1994). During cellular transformation, such as during embryonic development, it is not the genomic sequence but gene expression and related biological programs that change. In the embryo, changes in gene expression favor the maturation of the cell toward a certain cell lineage. Likewise, the phenotypic characteristics of cells can determine how they respond to external stimuli during their transformation into cancer cells. Our recent findings revealed that the “energy phenotype” of tumor cells drives their adaptive response to microenvironmental stress (Gnocchi et al. 2023a, 2023b, 2023c). In normal conditions, glycolysis, the TCA cycle, and OxPhos work together in a coordinated manner to maintain the cell’s quiescent state and prevent proliferation, which, according to Sonnenschein and Soto, is the cell’s default state (Soto and Sonnenschein 2016). This orderly functioning of eukaryotic cells can be altered, for example, by oxygen deficiency, excess glucose, or both. These conditions can be caused by inflammation, tissue damage, atherosclerosis, and fibrosis, which are more prevalent in certain locations of the body and are triggered by various risk factors for cancer, including a sedentary lifestyle, an unbalanced diet, metabolic syndrome, obesity, and diabetes. The TOFT made a significant contribution to the development of explanatory models of cancer by proposing that cancer is primarily a problem of tissue organization. This perspective shifts the dominant paradigm established by the SMT from a molecular level to a tissue level (Soto and Sonnenschein 2004). By acknowledging that alterations in tissue structure are important contributing factors to carcinogenesis, the SETOC posits that the disruption of endosymbiosis serves as the mechanism driving dedifferentiation and uncontrolled cell proliferation in cancerous tissues. To survive energetically, eukaryotic cells boost their glycolytic activity, generating more ATP, which also produces more waste in the form of lactate, a disposal problem that neighboring cells help solve partially by utilizing the lactate. Additionally, lactate is reused to synthesize molecules through anabolic pathways, which facilitates lactate elimination (DeBerardinis and Chandel 2020). Under hypoxic conditions, the TCA cycle and OxPhos are unable to function normally, resulting in limited ATP production, which prevents eukaryotic cells from maintaining a proper differentiation state (Lin et al. 2006; Fuhrmann et al. 2017). Under these conditions, cells are more likely to proliferate, become transformed, and eventually become malignant, migrating from neighboring cells (invasive cells) (Ortmann et al. 2014; Rankin et al. 2016). When mammalian cells undergo neoplastic transformation, another process that can be triggered is the reverse TCA cycle, which is induced by impaired energy metabolism and dysfunctional OxPhos (Filipp et al. 2012; Bezwada et al. 2024). The reverse TCA cycle, operating in certain bacteria and archaea, consists of a series of biochemical reactions that convert carbon dioxide and water into carbon compounds via energy-rich reductants such as electrons. For example, westernized diets, which typically consist of 70–80% high-glycemic index carbohydrates at high loads, can compromise the cell's integrated endosymbiotic systems when glucose levels increase and remain consistently high in the tissue microenvironment. In this case, lactate production increases, even when oxygen is present because glycolysis accelerates due to the abundance of glucose, the starting metabolite. When local inflammation and other pathogenic factors occur simultaneously, the respiratory chain and TCA cycle can become dysfunctional, leading to the TCA cycle operating in reverse and promoting the formation of cell proliferation products. Unfortunately, this condition is a response to cancer risk factors, such as an unbalanced diet and hyperinsulinemia, which are most common in Western societies and lead to an increase in cancer rates, even in younger individuals (Bray et al. 2024). Macro-level determinants, such as dietary habits, physical inactivity, and stress, can lead to a physiological shift toward a cancer-prone state, which includes conditions such as metabolic syndrome, obesity, and diabetes. Localized causal factors can then interact synergistically with these macro-level determinants. For example, in the colon, specific dietary components, toxins, chemical agents, or a dysfunctional gut microbiome can alter the microenvironment. This flow of causality leads to cellular adaptability and the impairment of endosymbiosis (de-endosymbiosis) as a response. Thus, de-endosymbiosis can be seen as the underlying common mechanism that responds to pro-carcinogenic insults. Endosymbiosis is necessary for the correct energetic functioning of eukaryotic cells. Thus, its impairment leads to an increase in glycolytic phases, lactate production via fermentation/aerobic glycolysis, a functional block of the respiratory chain, the reversal of the TCA cycle, and altered cellular communication at the cellular level. The remarkable plasticity of cancer cells, which allows them to exploit de-endosymbiosis, underpins these processes. Cellular adaptability and plasticity can be observed not only in cancers, which are typically characterized by dedifferentiation and an increased proliferation rate but also in pre-cancerous conditions such as dysplasia. In summary, the development of cancer is a result of local cellular evolution in response to changes in the tissue environment, which is also influenced by an organism's overall homeostatic-metabolic structure. The SETOC highlights the energetic dysfunction in eukaryotic cells, which results from de-endosymbiosis and is triggered by cancer-promoting factors such as chronic inflammation, hypoxia, and persistently elevated glucose levels.

### Consequences of the hypothesis and discussion

Cellular cognition enables cells to sense and adapt to changes in their microenvironment (Shapiro 2021). For example, cells adapt and respond to harsh microenvironmental conditions by adjusting their settings through biological processes and functions that are not typically used to maintain their differentiated state. The genetic programs coding these processes are dormant and often phylogenetically and ontogenetically preserved in cells. These programs promote anabolic and dedifferentiated states of the cell, which favors uncontrolled growth. Fermentation and the reverse TCA cycle are examples of phylogenetically conserved programs that cancer cells exhibit to adapt to their microenvironment. Cancer cells can also generate energy through fumarate respiration, a process commonly found in certain bacteria, in which fumarate takes the place of oxygen as an electron acceptor (Hirayama et al. 2009; Kröger et al. 2002). This suggests that cancer utilizes ancient metabolic programs similar to those found in single-celled organisms. During carcinogenesis, changes in the microenvironment trigger a shift to aerobic glycolysis, producing lactate, an ancient form of energy supply, which allows cells to exploit preserved phylogenetic programs and mimic the behavior of certain anaerobic unicellular organisms, regardless of OxPhos activity (Gnocchi et al. 2023b, 2023c).

**Fig. 1:**
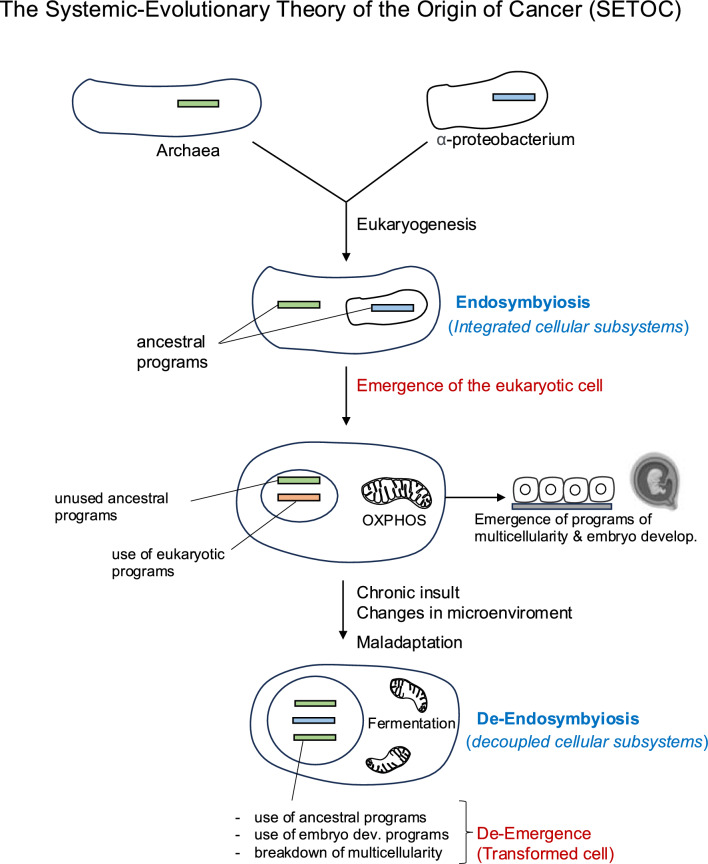
The systemic evolutionary theory of the origin of cancer (SETOC) views the process of neoplastic transformation as a dynamic adaptation of tissue cells to persistent pathogenic conditions. The fusion of primitive archaea and α-proteobacteria gave rise to the first common ancestor of eukaryotes (eukaryogenesis). During carcinogenesis, the archaeal component of the eukaryotic cell gradually functionally decouples from the α-proteobacterial component in response to chronic insults and environmental changes. The ability of the cell to adapt to harsh environments is made possible by the preservation of ancient mechanisms through evolution. Carcinogenic microenvironments can mimic the ancient environments that existed on Earth millions of years ago, environments in which cells coped. Continuous adaptation attempts and transitional states of de-endosymbiosis can ultimately result in maladaptation. As a consequence, the emerging system is the transformed cell (de-emergence) with a greater capacity to adapt to the changed conditions of the microenvironment

Similarly, the generation of embryonic markers and maintenance of a stem cell-like phenotype are examples of ontogenetic programs at work. The return to phylogenetic and ontogenetic characteristics is often seen as an evolutionary conversion to an ancestral state. Indeed, these features are present in cells but are not expressed under normal conditions. Therefore, the so-called atavism or evolutionary throwback is a change in the cell’s functional mode that creates a new thermodynamic attractor, enabling it to express a more robust core and better cope with certain microenvironmental conditions. That is to say, cells rely on remarkably resilient and evolutionarily conserved programs to survive in a hostile microenvironment. Vestiges emerge as a result of de-endosymbiosis. The SETOC suggests that during periods of cellular maladaptation, cells utilize ancient, evolutionarily conserved programs similar to those found in unicellular organisms (Fig. 1). During maladaptive cycles, cells can inappropriately activate embryonic morphogenetic programs, which are normally silenced. Although the term atavism is widely accepted, it should not be interpreted as a literal reversion to an ancestral state; instead, it represents an adaptive strategy in which cells adopt functional modules, ancestrally conserved or derived from embryonic life, that are better suited to the altered microenvironment. These functional modules confer robustness to tumor cells.

**Fig. 2:**
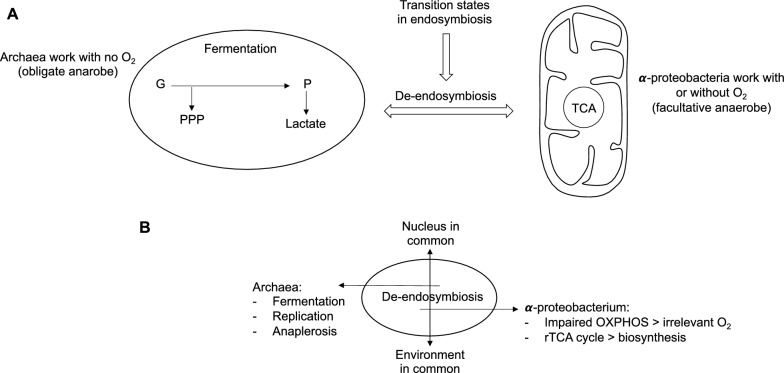
**A** De-endosymbiosis is characterized by different transition states, where the archaeal part, an obligate anaerobe, does not require O_2_, whereas the α-proteobacterial part is a facultative anaerobe. **B** The archaeal part and the α-proteobacterial part, the two endosymbiotic cellular subsystems, share a common nucleus and environment. During the gradual de-endosymbiosis, the two subsystems decouple, with one driving fermentation, replication, and anaplerotic reactions, and the other responsible for the dysfunction of OXPHOS and the (reverse) TCA cycle

During de-endosymbiosis, the cell exhibits distinct metabolic states, with the archaebacterial part functioning obligately anaerobically while the α-proteobacterial part operates facultatively anaerobically (Fig. 2A). Thus, the archaebacterial part (the cellular core) ferments, and shifts toward anabolism through pathways such as the pentose phosphate pathway and the glyoxalase system, entering a proliferative state. Instead, the α-proteobacterial part (the mitochondria) exhibits dysfunctional OxPhos, where the presence of oxygen can be irrelevant, leading to the reverse TCA cycle and anaplerotic reactions (Fig. 2B). The result is that tumor cells primarily generate energy through oxygen-independent anabolic processes to maintain a proproliferative state and adapt to changing environments. Although oxygen-dependent pathways may be present in certain cases, depending on the activity of the α-proteobacterial component.

The mechanism of de-endosymbiosis proposed by the SETOC can account for the ability of cancer cells to co-express aerobic glycolysis and OxPhos to varying degrees, a phenomenon known as cellular plasticity, which enables them to adapt to specific environments. Cancer cell plasticity refers to the reversible phenotypic and molecular changes in cancer cells that enable them to adapt to dynamic microenvironmental conditions (Niu et al. 2024). Recent studies indicate that cancer cells exhibit intrinsic plasticity, which may allow them to revert to a benign state, thereby challenging traditional views of cancer as an irreversible process (Pensotti et al. 2023). This intrinsic plasticity contributes to tumor heterogeneity and therapy resistance, enabling cancer cells to evade treatment (Niu et al. 2024). The SETOC suggests that tumor heterogeneity may arise from the presence of cells within a given tumor that exist in various stages of de-endosymbiosis, representing transitional phases. During de-endosymbiosis, most metabolic processes shift toward fermentable fuels and anabolic pathways. Fermentation is essentially a process that generates energy in the absence of oxygen. Although tumor cells can consume oxygen through OxPhos activity, which is often less energy-efficient, their fermentation rate increases at the expense of OxPhos when microenvironmental stressors are present (Gnocchi et al. 2023a; Gnocchi et al. 2022). Indeed, the heterogeneity of cancer explains why some respiratory activity can be detected and why its effectiveness varies. Despite the high rate of aerobic glucose metabolism, some tumors can produce energy through mitochondrial activity and thus ferment and oxidize glucose simultaneously (DeBerardinis and Chandel 2016; Koppenol et al. 2011). However, most importantly, cancer cells reprogram their metabolism, reversing the TCA cycle to generate energy for their growth. For example, in certain forms of cancer, lactate fermentation does not necessarily coexist with the downregulation or impairment of OxPhos (Diers et al. 2012; Viale et al. 2014). Furthermore, breast and colon cancer cells can reprogram lactate, converting it into pyruvate, which in turn supports the mitochondrial OxPhos (Diers et al. 2012; Kaldma et al. 2014; Chekulayev et al. 2015). The ambivalence of energy metabolism in tumors can be attributed to varying degrees of incoordination between the two subsystems that occur during de-endosymbiosis, resulting in different cell transition states during malignant transformation. Similarly, these de-endosymbiotic transition states can account for the competition between glycolysis and fatty acid oxidation, a phenomenon exploited by certain cancer cells and evaded by others (De Oliveira et al. 2020). This competition for energy sources is regulated by the glucose-fatty acid cycle, also known as the Randle cycle (Hue et al. 2009). The decoupling of the two subsystems during de-endosymbiosis can also manifest as a dysfunction in mitonuclear communication. Mitochondrial-nuclear communication is crucial in cancer biology, as it affects tumor development, stemness, and metabolic reprogramming (Mello et al. 2019; Fan et al. 2023). This interaction between mitochondria and the nucleus is essential for maintaining cellular homeostasis and can significantly impact cancer progression and treatment resistance (Singh 2024).

**Fig. 3:**
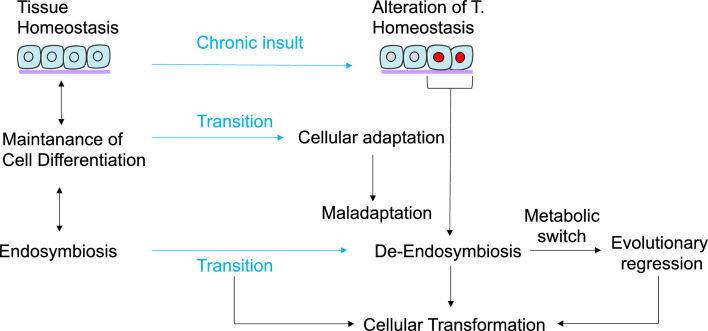
Cellular transformation, as described by the SETOC, involves a gradual and dynamic adaptation of tissue cells to a persistent insult, which disrupts homeostasis and causes tissue damage. This gradual dynamic adaptation is driven by the transition of both endosymbiotic cellular subsystems towards a de-endosymbiotic state, which results in a metabolic switch, the re-expression of ancient conserved biological processes, and cellular transformation

The question now arises naturally. What determines the endosymbiosis? Let us imagine a persistent high-amplitude insult that is difficult to dampen. If such an insult exceeds the cell’s exposure threshold, it can trigger a transition state of the cell itself that enables it to adapt to the new conditions. Over time, this mechanism eventually establishes a positive feedback loop that keeps the cell in a new state different from the initial one. Cellular de-endosymbiosis is the general mechanism that drives this process (Fig. 3). As a result of the mechanism of de-endosymbiosis, cells can adapt more effectively by transitioning to a new cellular organization and utilizing pre-existing programs stored in evolutionary memory during cellular transformation.

Microenvironmental conditions, such as hypoxia or anoxia, altered nutrient diffusion, or acidity, can disrupt cell homeostasis at the tissue level, making the ecosystem unsustainable for the cell. These conditions are somewhat similar to those found billions of years prior to the presence of oxygen on Earth. Because cells have interacted with these environments during their evolutionary history, they have retained adaptive information in their genome and can cope with these conditions. From an evolutionary perspective, cells that fail to adapt cannot retain this adaptive information because they cannot survive. Thus, only the information that survived in that environment has been preserved and passed from one generation to the next (primarily information from unicellular organisms, not multicellular organisms). From an evolutionary perspective, cancer is primarily the result of cells adapting to a changed tissue environment, leading to maladaptation, rather than being caused by genetic mutations in cancer cells, which are merely secondary downstream effects (Mazzocca et al. 2016; Mazzocca et al. 2021; Gnocchi et al. 2023a). This maladaptation allows cancer cells to survive in an altered environment by exploiting the activation or deactivation of genes, particularly ancient genes (Trigos et al. 2017), as well as the rewiring of metabolic programs unrelated to genes. Cells possess memory, sentience, and adaptability, which allows them to survive in response to challenging environmental stimuli. Ultimately, this adaptation becomes maladaptive, compromising the entire cellular system and leading to cancer (Gnocchi et al. 2023a, 2023b; Gnocchi et al. 2023a, b, c).

### Conclusive remarks

The origin of cancer does not stem from defects in individual cellular components, such as the nucleus, DNA, cytoplasm, mitochondria, or other organelles, but rather from defects in communication between cellular subsystems, as outlined in the de-endosymbiosis theory proposed by the SETOC. Therefore, the problem lies in miscommunication, which is a defect in how parts interact and integrate. The SETOC suggests that the outcome of numerous adaptations to increasingly hostile microenvironmental conditions is a communication defect between two endosymbiotic cellular subsystems, enabling cells to thrive in a new environment through a robust mechanism. The insult should be intense enough to persist over time but not so strong that it causes the cells to die. For instance, this condition is observed in dysmetabolic or inflammatory states, where the microenvironment undergoes changes, such as alterations in metabolite, cytokine, and growth factor concentrations, or in cases of fibrosis, where the extracellular matrix is altered. The cell responds with a positive feedback mechanism that adapts through de-endosymbiosis. Cells adapt to changing environments by relying on de-endosymbiosis to varying degrees depending on their needs during transformation. Hence, the degree of cellular dedifferentiation and aberrant metabolism (e.g., fermentation versus OxPhos) is determined by the extent of de-endosymbiosis achieved under specific conditions. As the SETOC suggests, carcinogenesis is an adaptive process, making it conceivable that both preventive and therapeutic interventions could be possible. One potential intervention is to preserve a healthy microenvironment by preventing cellular-level damage (at the meso-level, e.g., local inflammation) and molecular-level harm (at the micro-level, e.g., high blood sugar and insulin levels, cytokines, and growth factors). The macro-level involves the interaction between the organism and the environment, influencing the meso- and micro-levels, such as unbalanced diets, sedentary lifestyles, stress, and toxins. Lifestyle changes and lifestyle medicine are important interventions at the macro-level. Such interventions can also increase the efficacy of standard anticancer therapies.

## Data Availability

No datasets were generated or analysed during the current study.

## Abbreviations

ATP: Adenosine triphosphate
DNA: Deoxyribonucleic acid
OxPhos: Oxidative phosphorylation
SETOC: Systemic evolutionary theory of the origin of cancer
SMT: Somatic mutation theory
TCA: Tricarboxylic acid
TOFT: Tissue organization field theory of cancer
